# Parental methyl-enhanced diet and in ovo corticosterone affect first generation Japanese quail (*Coturnix japonica*) development, behaviour and stress response

**DOI:** 10.1038/s41598-021-99812-w

**Published:** 2021-10-26

**Authors:** Kay Boulton, Peter W. Wilson, Valerie R. Bishop, Jonathan H. Perez, Toby Wilkinson, Kris Hogan, Natalie Z. M. Homer, Christelle Robert, Jacqueline Smith, Simone L. Meddle, Ian C. Dunn, Kellie Watson

**Affiliations:** 1 The Roslin Institute and R(D)SVS, The University of Edinburgh, Easter Bush, Midlothian, EH25 9RG UK; 2 Centre for Cardiovascular Sciences, Mass Spectrometry Core, E3.08, Queen’s Medical Research Institute, The University of Edinburgh, 47 Little France Crescent, Edinburgh, EH16 4TJ UK; 3 Department of Biology, University of South Alabama, Mobile, AL 36688 USA

**Keywords:** Epigenetic memory, Behavioural ecology, Evolutionary developmental biology, Neurophysiology, Stress and resilience

## Abstract

The role of maternal investment in avian offspring has considerable life history implications on production traits and therefore potential for the poultry industry. A first generation (G_1_) of Japanese quail (*Coturnix japonica*) were bred from a 2 × 2 factorial design. Parents were fed either a control or methyl-enhanced (HiBET) diet, and their eggs were treated with a vehicle or corticosterone injection during day 5 of incubation. A subset of G_1_ birds were subjected to an open field trial (OFT) and capture-restraint stress protocol. Significant effects of HiBET diet were found on parental egg and liver weights, G_1_ hatch, liver and female reproductive tract weights, egg productivity, latency to leave the OFT central zone, male baseline 11-dehydrocorticosterone, and female androstenedione plasma concentrations. In ovo treatment significantly affected latency to return to the OFT, male baseline testosterone and androstenedione, and change in androstenedione plasma concentration. Diet by treatment interactions were significant for G_1_ liver weight and male baseline plasma concentrations of corticosterone. These novel findings suggest significant positive effects on reproduction, growth, precociousness, and hypothalamic–pituitary–adrenal axis function from enhanced methyl diets, and are important in understanding how in ovo stressors (representing maternal stress), affect the first offspring generation.

## Introduction

The role of maternal investment, especially nutritional, in avian offspring has considerable life history implications on production and therefore potential for the poultry industry, and has been well documented^1–4^. Transmission of non-genetic effects to offspring may vary depending on the age of the mother^5^, while antibody transfer to eggs is related to maternal condition^6^. Maternal environmental exposure can result in epigenetic modification of gene expression by DNA methylation^7^, with transgenerational inheritance of epigenetic variation a possibility^8–10^. There are many examples of studies suggesting that uniformly beneficial epigenetic changes can be induced by enhancing consumption of essential dietary nutrients, summarized in^11^.

Maternal nutritional biochemistry may be linked to DNA methylation through dietary changes in levels of the essential nutrients—folate, vitamins B_2_, B_6_ and B_12_, choline, betaine and methionine—required for 1-carbon metabolism^12–14^, especially in early life^15^. 1-carbon metabolism, the series of interlinking metabolic pathways that are central to cellular function, provides methyl groups for the synthesis of amino acids, creatine, DNA, phospholipids, and polyamines^12,13^. Acting as a methyl donor to the 1-carbon metabolism pathway, betaine, a trimethyl derivative of the amino acid glycine, can substitute for methionine and choline in amino acid production, and hence, protein and lipid synthesis.

As poultry cannot synthesize the methyl group, the practice of adding purified betaine as a dietary supplement to poultry feed is known to produce many benefits^16–18^. In its capacity as an organic osmolyte, betaine offers an immunological role, supporting intestinal growth by protecting epithelial cells from environmental stress, e.g. coccidial infection, and promoting intestinal microbiota population^16,19–21^. Betaine also potentially influences the digestibility of nutrients, thus enhancing meat quality and carcass composition, bone strength, egg quality and egg production in poultry^16,18,21–27^.

The prolonged effects of stress exposure during prenatal development on animal physiology and behaviour is well documented in avian species including zebra finch (*Taenopygia guttata*), Japanese quail (*Coturnix japonica*), and the domestic chicken (*Gallus gallus*)^28–31^. Importantly, early life stress in food producing animals, especially heat stress in poultry species, can have detrimental effects on meat quality^32^. The hypothalamic–pituitary–adrenal (HPA) axis is activated during novel and stressful situations, with the release of glucocorticoids enabling a rapid biological response that diverts behaviour to essential survival activities^33–36^. Whilst disruption of the HPA axis during chronic stress is indicative of detrimental effects^37^, the rapid return of glucocorticoids to baseline plasma concentrations facilitates additional adaptive risk-taking behaviours and may allow better coping strategies^38–40^.

Experimental pre-natal manipulation of the HPA axis is possible in avian species via dietary and in ovo transfer of glucocorticoids^37,41^. The injection of corticosterone into quail eggs during early incubation has been demonstrated to promote increased activity and exploration levels in a novel environment through dilution of physiological responses^42^.

In this study, we tested the effects of parental betaine-enhanced diet and an in ovo HPA axis manipulation (parental stressor simulation) on growth and behaviour in a first generation (G_1_) of Japanese quail. Quail were used due to their short generation interval, the avoidance of confounding in utero post-hatch maternal effects, and ease of housing and handling in a commercial rearing facility^43^. A high betaine diet was selected to facilitate the generation of methionine from homocysteine^18^. We used a 2 × 2 factorial design to create four study groups: control diet with vehicle (−/−); betaine supplemented diet with vehicle (+ /−); control diet with in ovo corticosterone treatment (−/+); betaine supplemented diet with in ovo corticosterone treatment (+/+), outlined in Table 1. We hypothesised that an enhanced betaine parental (G_0_) diet would have a positive effect on G_1_ growth and development, with detrimental effects on behaviour and stress response from subjecting G_0_ eggs to corticosterone treatment during development. We also anticipated possible enhanced effects of diet by treatment interaction (diet*treatment) on growth, development, and stress response.

**Table 1:** Experimental 2 × 2 factorial design representing the number of G_1_ individuals in each category with complete data sets; Diet/treatment key: − = no diet or treatment applied, +  = diet or treatment applied.

	G_0_ Diet		
In ovo treatment	Control	Enhanced Betaine (HiBET)	Total
Control (Vehicle)	−/− 55	−/+ 49	104
Corticosterone suspended in vehicle	+/− 41	+/+ 45	86
Total	96	94	190

## Results

### Growth and productivity

**Figure 1:**
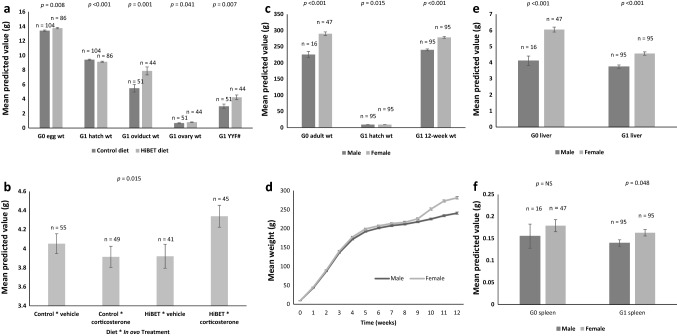
Growth and productivity of G_0_ and G_1_ quail. Significance (*p*) values are indicated for each trait. (**a**) Mean predicted value of parental diet effect on egg weight, G_1_ chick hatch weight, G_1_ oviduct weight, G_1_ ovary weight (g; ± s.e.m.) and G_1_ yellow yolked follicle number (YYF #; ± s.e.m.). HiBET = betaine enhanced diet. (**b**) Mean predicted value of diet by in ovo treatment interaction for G_1_ liver weight (g; ± s.e.m.). (**c**) Mean predicted value of sex for G_0_ body weight, G_1_ hatch weight and G_1_ 12 week weight (g; ± s.e.m.), where Wt = weight. (**d**) Unadjusted mean G_1_ body weight (g) ± s.e.m. from hatch to twelve weeks. (**e**) Mean predicted value of sex on G_0_ and G_1_ liver weight (g; ± s.e.m.). (**f**) Mean predicted values of sex on G_0_ and G_1_ liver, and G_1_ spleen weight (g; ± s.e.m.).

Significant positive effects of G_0_ diet were seen on the mean weight of G_0_ eggs carrying G_1_ embryos, with betaine enhanced diet (HiBET) fed females laying heavier eggs than females fed the control diet (Egg_wt_G0_HiBET_ =  + 0.35 ± 0.13 g, *p* = 0.008; Fig. 1a; Supplementary Table S3). HiBET had a significant negative effect on G_1_ hatch weights, with chicks from HiBET fed G_0_ parents being lighter than those from control fed parents (Hatch_wt_HiBET_ = 0.28 ± 0.07 g, *p* < 0.001; Fig. 1a; Supplementary Table S3). Egg, hatch weight, and 12-week weight were all positively correlated (*p* < 0.05; Supplementary Table S4).

The HiBET diet had positive effects on G_1_ productivity as indicated by significantly heavier mean oviduct and ovary weights after adjusting for body weight (oviduct_HiBET_ = 2.50 ± 0.76 g, *p* = 0.001; ovary_HiBET_ = 0.12 ± 0.06 g, *p* = 0.041; Fig. 1a; Supplementary Table S3), and the mean number of yellow-yolked follicles present (YYF_HiBET_ = 1.24 ± 0.44, *p* = 0.007; Fig. 1a; Supplementary Table S3). There were significant correlations between the three traits (*p* < 0.05; Supplementary Table S4). Additionally, a higher percentage of females from control fed parents (mean 35.3%) were out of lay at 12 weeks compared with those from the HiBET fed parents (mean 6.8%; Supplementary Table S5). There was no significant effect of G_0_ diet on G_1_ testes weight.

A diet by in ovo treatment interaction (diet*treatment) for G_1_ liver weight was significant, with those quail from HiBET parents receiving the corticosterone treatment (+/+) having heavier livers than the other categories (liver_HiBET*B_ =  + 0.56 ± 0.23 g *p* = 0.015; Fig. 1b; Supplementary Table S3). Otherwise, in ovo treatments with corticosterone were not significant for G_1_ growth or organ weights, and no interactions between sex and diet or in ovo treatment were evident for growth traits.

Although G_1_ female chicks were heavier than males (F_chick_ =  + 0.16 ± 0.07 g, *p* = 0.015; Fig. 1c; Supplementary Table S3), there was no evidence of diet by sex interaction (diet*sex) on mean chick weight. By adulthood, females from both G_0_ and G_1_ were significantly heavier than their counterpart males (G_0__WT_F_ =  + 64.3 ± 10.98 g, *p* < 0.001; G_1__12WK_WT_F_ =  + 38.6 ± 4.20 g, *p* < 0.001; Fig. 1c; Supplementary Table S3). Indeed, G_1_ females were heavier than G_1_ males throughout the trial (Fig. 1d).

After adjusting for body weight, females from both generations had significantly heavier livers (G_0__Liver_F_ =  + 1.94 ± 0.36 g, *p* < 0.001; G_1__Liver_F_ = 1.14 ± 0.15 g, both *p* < 0.001; Fig. 1e; Supplementary Table S3). Similarly, G_1_ female spleens were also significantly heavier than those of the G_1_ males (G_1__Spleen_F_ =  + 0.02 ± 0.01 g, *p* = 0.048; Fig. 1f; Supplementary Table S3), and although the unadjusted mean female G_0_ spleen weight was heavier than that of the G_0_ male, following statistical analysis this was not significant. There were no significant effects of G_0_ diet or sex by diet interaction (sex*diet) on final body, liver spleen weights of either the G_0_ or G_1_ quail. G_1_ spleen weights were significantly correlated with both G_1_ male and female reproductive organ weights, while liver weights were not (Supplementary Table S4).

### Behaviour

Parental diet had a significant effect on latency to move (LtMove) after entering the OFT arena. Although the majority of the birds moved very quickly after being placed in the arena, of those that remained stationary for longer, the G_1_ from parents fed the HiBET diet moved faster (Log_e__LtMove_HiBET_ = − 0.69 ± 0.27 s, *p* = 0.019; Supplementary Table S6). G_1_ quail from eggs treated with corticosterone (B) were significantly faster to revisit the middle zone after initial positioning (Log_e_ _LtVMZ_B_ = − 1.24 ± 0.58 s, *p* = 0.038; Supplementary Table S6).

Females were significantly slower to visit the outer zone (Log_e_ _LtVOZ_F_ =  + 1.72 ± 0.67 s, *p* = 0.010), paid fewer visits to it (#VtOZ_F_ = − 3.15 ± 1.52, *p* = 0.043), and spent less time there than males (TiOZ_F_ = − 44.3 ± 19.7 s, *p* = 0.029; Supplementary Table S6). Conversely, females also paid significantly fewer visits to the middle zone (#VtMZ_F_ = − 3.41 ± 1.35, *p* = 0.015; Supplementary Table S6), and although they spent longer there than males (time in middle, TiMZ), this latter trait was not significant.

Females travelled significantly shorter distances than males (Log_e_ _D_F_ = − 0.50 ± 0.20 cm, *p* = 0.019; Supplementary Table S6) and at slower velocities (V), (Log_e_ _V_F_ = − 0.43 ± 0.21 cm/s, *p* = 0.019; Supplementary Table S6). Females also spent significantly less time moving than males (TMov_F_ = − 31.8 ± 14.8 s, *p* = 0.031; Supplementary Table S6). High correlations exist between the numbers of visits to the middle zone, time spent moving, distance travelled, and velocity of movement (Supplementary Table S7).

Females were significantly slower to commence scratching the ground (Log_e_ _LtScratch_F_ = − 0.48 ± 023 s, *p* = 0.034), and also spent less time doing so than males, (Tscratch_F_ = − 40.3 ± 18.2 s, *p* = 0.033; Supplementary Table S6).

### Circulating steroid hormones

**Figure 2:**
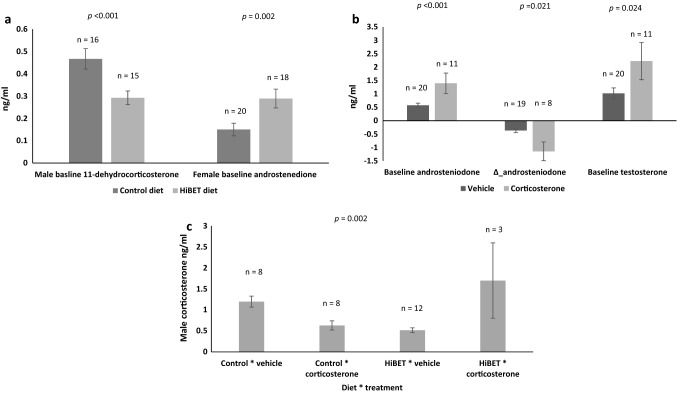
Circulating G_1_ steroid hormones. Significance (*p*) values are indicated for each trait. (**a**) Unadjusted raw data for effect of parental diet (± s.e.m.) on G_1_ male baseline level of 11-dehydrocorticosterone, and G_1_ female baseline plasma concentration of androstenedione. HiBET = betaine enhanced. (**b**) Unadjusted raw data for effect of in ovo treatment (± s.e.m.) on male baseline and change (Δ) in androstenedione plasma concentrations following stressor, and baseline plasma concentration of testosterone. (**c**) Unadjusted raw data for effect of parental diet by in ovo treatment interaction (Diet*treatment) (± s.e.m.) on G_1_ male baseline plasma concentration of corticosterone.

Parental diet had a significant effect on G_1_ estimated Log_e_ mean baseline (base) 11-dehydrocorticosterone plasma concentration, with males from HiBET fed parents having lower plasma concentrations than those from control fed parents (Log_e_ _base_11-dehydrocorticosterone_HiBET, M_ = − 0.47 ± 0.12 ng/ml, *p* < 0.001, Fig. 2a; Supplementary Table S8). Estimated female mean baseline androstenedione plasma concentration was also significantly affected by parental diet, showing increased baseline plasma concentration (base_androstenedione_HiBET, F_ =  + 0.13 ± 0.04 ng/ml, *p* = 0.002; Fig. 2a; Supplementary Table S8).

Corticosterone (B) in ovo treatment significantly affected only male steroid hormone plasma concentrations, with estimated Log_e_ baseline plasma concentrations for androstenedione and testosterone being significantly higher than for those receiving the control treatment (Log_e_ _base_androstenedione_B, M_ =  + 0.51 ± 0.14 ng/ml, *p* =  < 0.001; Log_e_ base_T_B, M_ =  + 0.79 ± 0.32, *p* = 0.021; Fig. 2b; Supplementary Table S8). Changes in androstenedione plasma concentration after stress were lower in those males from eggs treated with corticosterone (Δ_androstenedione_B, M_ = − 0.14 ± 0.06 ng/ml, *p* = 0.24; Fig. 2b; Supplementary Table S8).

A parental diet by in ovo treatment interaction (diet*treatment) was seen for baseline plasma concentration of corticosterone, again only in males, with those males from the HiBET fed parents that received the in ovo treatment having significantly higher estimated baseline concentration than the other categories (Log_e_ _base_B_HiBET*B, M_ =  + 1.49 ± 0.44 ng/ml, *p* = 0.002; Fig. 2c; Supplementary Table S8).

## Discussion

In this study we have determined the effects of a parental (G_0_) methyl-enhanced diet and a simulated maternal stressor on growth, maturation, behaviour and stress in a single subsequent quail generation (G_1_).

Eggs laid by G_0_ females fed an enhanced diet (HiBET) were significantly heavier than those fed a control diet, similar to findings in previous studies^17,26,44^. Hepatic enzyme activity is known to be enhanced when poultry diets are supplemented with betaine, resulting particularly in the mobilisation of cholesterol from stored abdominal fat to tissue oxidation and metabolism^22,27^. Increased egg yolk fat content has been found when hen diets have been supplemented with the essential amino acids methionine, choline or betaine^17,45,46^. As methionine is understood to be the first limiting essential amino acid in laying birds^22^, its function as a methyl donor can be spared if betaine is present in the diet. We suggest that higher levels of cholesterol deposits in yolks may be contributing to increased egg weights from our HiBET females. Additionally, it is possible that some of the increased HiBET egg weight is due to thicker (and therefore heavier) eggshell, as reported in some studies using methionine supplemented diets^47,48^. Chicks from eggs of G_0_ HiBET females were significantly lighter than those from control fed G_0_ females, and while this is contradicted in some previous reports^44^, adds weight to our suggestion.

While parental diet had no effect on final body weights of either the G_0_ or G_1_, also in line with previous studies^44^, G_1_ oviducts of females from HiBET parents were significantly heavier than those from the control fed parents. Our study also found enhanced baseline plasma concentrations of androstenedione in HiBET females. Androstenedione is an endogenous weak androgen steroid that is intermediate in the production of estrone, a weak oestrogen compound, following conversion by aromatase^49^. Early experiments on estrone injections in young female White Leghorn chicks resulted in rapid growth of the genital tract^50^. Although our study design did not allow for birds to be housed in treatment groups, or recovery of oviduct tracts prior to twelve weeks, this result may be indicative of earlier sexual maturity onset. Alternatively, the enhanced androstenedione plasma concentrations in these birds could be symptomatic of a stronger HPA axis drive. The correlation between oviduct weight and numbers of follicles present was high, with a positive effect on G_1_ productivity from the HiBET diet. Moreover, considering the higher percentage of sexually regressed females and lower yield per bird from control fed parents, there are very likely to be positive downstream methylation implications for sexual maturity and productivity from the HiBET diet^51,52^. Indeed, other studies have demonstrated that 1-carbon-metabolic essential nutrient diet supplementation can heighten reproductive performance^23,46,47^. It is plausible that these effects are again due to increased hepatic enzyme activity that has been transmitted via transgenerational mechanisms to the G_1_, resulting in (at least) enhanced reproductive performance.

HiBET offspring were faster to move after entering the open field trial arena. This could be interpreted as the quail exhibiting a higher level of anxiety and therefore seeking shelter from the outer wall of the arena. HiBET males also had reduced baseline plasma concentration of 11-dehydrocorticosterone, a precursor to corticosterone production. However, de novo synthesis of 11-dehydrocorticosterone can occur directly from cholesterol^53^. It is possible that the presence of 11-dehydrocorticosterone in the plasma of the HiBET birds is indicative of systemic regulation, acting as a pool for rapid generation of additional corticosterone as required by the liver.

In G_1_, in ovo treatment had a significant effect on latency to revisit the middle zone of the OFT arena, with those birds receiving in ovo corticosterone being slower to do so. Again, coupled with less exploratory behaviour and locomotion, this may be a demonstration of higher levels of anxiety in their novel surroundings. Androstenedione production in the in ovo corticosterone treated males was affected, with higher baseline plasma concentrations measured prior to the stressor, and consequently, less change afterward. Androstenedione is also an intermediate in the production of testosterone, and indeed, baseline plasma concentrations of these two steroid hormones are significantly correlated (Supplementary Table 7). Enhanced baseline plasma concentrations of corticosterone combined with increased baseline androstenedione and testosterone may contribute to the risk-taking behaviour of males in the OFT, especially given the positive correlations between these three steroid hormones.

There were no direct effects of in ovo treatment with corticosterone on growth, or reproductive organ weights of the G_1_. Livers were heavier in quail receiving the in ovo treatment from HiBET parents, and although livers in females are generally recognised to be heavier in laying females^51,52,54^, the correlation between liver and female reproductive organ weights was very low and not significant (Supplementary Table 4). Females from the +/+ group displayed a higher level of production than those from the other groups. A significant interaction between diet and treatment was also evident for G_1_ liver weights, with the in ovo treatment having a negative effect on liver weight from the control diet parents, and a positive effect on the birds from HiBET diet parents. Diet by treatment interactions were apparent for male baseline plasma concentrations of corticosterone. Liver weight could potentially be influenced by increases in hormonal activity, and while our study did not measure these hormones, an early study found evidence of inhibited liver enzyme activity in chickens fed enhanced betaine diets^55^.

Overall, females were heavier than males, with significant differences seen from hatch weight through to twelve weeks of age, and the onset of sexual maturity had a more marked effect on weight gains at nine weeks, (Fig. 1d). It is worth noting that a direct comparison between the G_0_ and G_1_ final body weights is not possible because the G_0_ were older than the G_1_ at the time of these data collection. As predicted, due to lipid production by the liver for incorporation into egg yolk under the influence of female steroid hormones^56^, females from both generations had heavier livers, and G_1_ females had heavier spleens than G_1_ males, with a significant correlation between the G_1_ spleen and liver weights. The correlations between G_1_ liver weight and female reproductive organ weights were not significant, and in the case of G_1_ males were also negative although still non-significant. However, there were significant correlations between G_1_ spleen weights and both G_1_ male and female reproductive organ weights. Also as predicted, there were significant correlations between liver, spleen and 12-week weights, as well as with and between egg and hatch weights.

No interactions between sex, diet or in ovo treatment were seen in the OFT trial. Females were slower to visit the outer zone in the OFT, made fewer visits to it, and spent less time there than males. Consequently, females spent more time in the middle zone, crossing the boundary less frequently, and did not scratch for food as frequently as males. When females did move, this was at slower velocity and shorter distances than males. Independent of sex, enhanced plasma concentrations of testosterone in Japanese quail have been demonstrated to influence displays of more exploratory behaviour that may explain the sex difference in our results^57^. Evidence from avian studies suggests that maternal environments affect the amount of steroid hormones deposited in yolks, resulting in maternally derived phenotypic variations in coping styles^58,59^, with sustained differences in overall morphology, physiology and behaviour.

In conclusion, we found significant effects of parental increased methyl diet and a simulated parental stressor on a first generation of offspring were apparent for several growth, reproduction, behaviour, and circulating steroid hormone traits. These novel phenotypic findings are an important first step in understanding maternal nutritional and steroid hormone investment that potentially includes genome methylation on the phenotypes of a first generation. Specifically, the high-betaine parental diet produced heavier eggs but lower hatch-weight chicks, more productive first generation females, and first generation offspring, with differing circulating baseline plasma concentrations of HPA axis hormones exhibiting higher levels of anxiety. The simulated parental stress treatment only directly affected male HPA axis circulating steroid hormones involved in testosterone and its production. Interactions between the two treatments were explicitly seen on offspring liver weight and male baseline plasma concentrations of corticosterone. Future work on a larger scale, including further generations and examination of methylation intensity and patterns in egg production should improve these findings, and are important to enhance the understanding of the mechanisms underpinning the transgenerational transfer of epigenetic effects in precocial avian species.

## Materials and methods

ARRIVE guidelines (https://arriveguidelines.org/arrive-guidelines) were followed at all stages of the trial.

### G_0_ production

A base population (generation 0, G_0_) of 100 Japanese quail (*Coturnix japonica*) chicks were produced at the National Avian Research Facility (NARF), using a line maintained at the facility (http://www.narf.ac.uk/chickens/lines.html). On day of hatch, the G_0_ chicks were distributed equally between one of two dietary treatment groups housed in separate pens. One group received a normal (control) diet (Supplementary Table 1; Target feeds: https://www.targetfeeds.com), with the other group receiving the same diet enhanced with 0.075% betaine (HiBET). The treatments were maintained throughout the trial. Pen size, temperature and photoperiod were followed in line with recommended UK DEFRA guidelines (https://www.gov.uk/government/publications/poultry-on-farm-welfare/poultry-welfare-recommendations; Supplementary Table 2), and quail were fed ad libitum. The birds were then maintained on a 14L:10D photoperiod; lights on: 07:00.

### G_1_ production

At eight weeks of age, 100 G_0_ quail were sexed from their plumage, and male numbers reduced to sixteen in total (eight per diet group). There were a total 24 females in the control and 23 females in the HiBET groups. Over the course of the next three weeks, eggs were collected daily. As the females were group housed it was not possible to identify egg pedigree. Eggs were washed with Rotosan Egg Wash Powder (https://www.solwayfeeders.com/housing-incubation-brooders/egg-washing/rotosan-egg-wash-powder/) and weighed to the nearest 0.01 g. Eggs from the two treatment groups were distinguished by different coloured pre-numbered (1 − n) 1 cm diameter circular sticky labels (Brady, cat. No. M71-89-499). Eggs were stored prior to incubation at 14.0 °C. At day seven of collection, all available eggs were placed laterally in a sterile incubator at 37.5 °C and 55% humidity. To avoid bias, eggs were positioned in sets of 4 × 4 as follows: each day’s collection from the HiBET or control pens were ranked, and then randomised on the basis of weight into two groups. This represented those eggs that were to receive an injection of corticosterone or a peanut oil vehicle at embryonic development day 5 (E5; see “In ovo treatments” section below), and thus created the 2 × 2 factorial design of + /− HiBET and + /− corticosterone generation 1 (G_1_), while simultaneously ensuring that the numbers in each group were approximately equivalent (Table 1). Multiples of eight eggs, based on weight, were designated as a batch, there being complementary batches for HiBET and control diet fed birds containing equal numbers of eggs to be injected with corticosterone or vehicle. These complementary batches were further randomised to avoid any effects of order of injection. Randomisation was generated using the = RAND() function in Microsoft Excel.

On the day prior to hatch (E16), eggs were placed in individual numbered poultry pedigree hatching boxes (77 × 65 × 77 mm; http://www.dwcases.co.uk/) and transferred to the hatching incubator (custom made, https://bristolincubators.com). After hatch (E17-18), chicks were removed from their boxes, weighed and leg ringed, with the box number cross-referenced to the leg ring number. Chicks were then returned to the hatching incubator for a few hours prior to transfer to a small rearing pen with a heat lamp, water and quail chick crumb. The chicks were then maintained on a 18L:6D photoperiod; lights on: 07:00.

At three days of age (D3), leg rings were removed and chicks were wing tagged. Chicks were returned to their rearing pen for a further two weeks, when they were transferred to standard housing pens. Three hatches of G_1_ birds were bred and reared in this way, one week apart. Each hatch was kept in a separate housing pen. In total, n = 190 G_1_ birds with complete sets of records were reared to sexual maturity. Only the quail from hatch_1_ (n = 69) were included in separate behaviour and stress challenges, performed at WK7 and WK11, respectively. The chicks were then maintained on a 10L:14D photoperiod; lights on: 07:00.

### In ovo treatments

In ovo treatments for the G_0_ eggs containing the G_1_ embryos were pre-prepared following Marasco et al.,^60^: An 850 ug /ml corticosterone (B) stock solution was made by suspending 0.085 g corticosterone (https://www.sigmaaldrich.com/catalog/product/sigma/) in 100 ml sterilized (i.e. autoclaved) peanut oil, sonicated in a water bath for several hours until dissolved. This was serially diluted to achieve the final concentration for injection of 850 ng/ml. The vehicle solution comprised sterile 100% peanut oil. Solutions were kept at room temperature and sonicated prior to use to disperse any cloudiness.

At day five of incubation (E5), 50 μl luer tipped Hamilton syringes were pre-prepared with corticosterone or vehicle solutions and air bubbles were dispersed. Eggs were removed from the incubator in the same batches described above. The apex of each egg was sanitised with 75% ethanol and a small hole was made using a fresh 25 G needle. The pre-prepared Hamilton syringe was inserted through the hole and 10 µl of either the corticosterone (dose: 8.5 ng) or vehicle was deposited into the yolk, and the hole sealed with a 2–3 mm square piece of Leukosilk (https://www.bsnmedical.com/products/wound-care-vascular/category-product-search/acute-wound-care/fixation/leukosilkr.html). Eggs were then returned to the incubator and placed apex down in the original locations. Only the handlers performing the injections were aware of the in ovo treatment experimental group.

### Open field trials

The open field trial (OFT) test protocol was adapted from the method of Satterlee and Marin^61^, a test of fearfulness, exploration and anxiety in Japanese quail. Commencing at seven weeks of age (WK7), Hatch_1_ were subjected to a single OFT carried out over the course of the next seven days, in three batches. The OFT arena comprised a 1 m^2^ pen made from four 1 × 1 m^2^ sheets of 10 mm birch plywood, secured with duct tape, and was placed inside an empty avian cage in and empty room. A Hikvision Digital Video Recorder DS-7732N1-SD (http://www.hikvisioniran.com/Hiwatch/DS-7600NI-SP.pdf) and integrated software was used to record the activity filmed by a HIKVISION IR NETWORK CAMERA DS-2CD2612F-I (https://www.hikvision.com/uploadfile/image/20150511064920320.PDF ).

The 69 quail from Hatch_1_ were randomly selected on a first-come-first-served basis in six groups of ten (plus one group of nine) from their housing pen and transferred in a 80 × 45 × 30 cm chicken crate to an experimental room where the crate was positioned on the floor and covered with a black rubber mat. Birds were selected at random, again on a first-come-first-served basis from the crate, positioned randomly (not pre-ordained) facing one of the four OFT arena sides to eliminate bias in direction of first movement, and filmed for a five minute period. After recording, sex was noted and a purple ring was placed around the left leg to ensure birds were only sampled once. After all ten birds had been tested, the procedure was repeated with a new batch of quail. All OFT were carried out by the same handler, who was blinded to experimental group.

### Stress trials

Commencing at WK11, over the course of three consecutive mornings, stress trials were carried out on Hatch_1_ only. Sampling followed a standardized capture-handling-restraint stress protocol adapted from Wingfield (1994)^35^. All sampling commenced at 09:00 following a 12 h period with no disturbance, and took place within three minutes of entering the room. On each occasion, the same handler entered the pen, captured, and passed birds individually to a second handler. Each bird was restrained while 100 μl blood was sampled from a brachial venepuncture, using a 25 G (0.5 mm) needle into heparinised 0.5 × 75 mm capillary tubes. Capillary tube content from each bird was transferred into a single 1.5 ml Eppendorf tube, labelled with the corresponding wing tag number. Samples were stored on ice prior to processing. Immediately following sampling, cotton wool was applied with pressure to the wound, and when bleeding had stopped, the bird was transferred to a 20 × 30 cm opaque cloth bag with a drawstring closure and restrained. At the end of the three minute period, any captured but unused birds were released back into the pen. The assembled restrained birds were transferred to the procedure room where they remained undisturbed for the following 30 min. After 30 min, birds were removed individually from their bags and a second blood sample was collected in the same manner described above. The purple leg tag applied during the earlier behaviour trials was removed, and the bird was then placed in a poultry crate. Once all the birds had been processed, they were returned to the pen. Removing the purple leg tags at this stage ensured that birds were captured and tested on a single occasion only. Plasma was separated within three hours of the procedure, by centrifugation (8000 g; 4 °C; 10 min), then removed to fresh tubes and stored at − 20 °C for future steroid hormone analysis.

### Growth and maturation phenotype collection

Body weights were collected from all 190 G_1_ quail on a weekly basis throughout the study. All handlers were blinded to experimental groups. Sex was noted by the appearance of secondary sexual characteristics at five weeks (WK5) of age. At twelve weeks of age (WK12), G_1_ birds were culled by cervical dislocation followed immediately by decapitation, and collection of 5 ml blood from the neck arteries. Testes were removed from males and weighed. Mature eggs were removed from females, prior to oviducts being weighed. A note was made of the number of yellow yolky follicles (YYF) present in the ovaries, and the ovaries minus the YYF were weighed. Livers and spleens were also removed and weighed. All samples were frozen on powdered dry ice and subsequently stored at − 80 °C until further analysis. The G_0_ were culled and blood was collected in the same way at the end of the egg collection period, with body and liver weights recorded and samples stored as described above for the G_1_.

### Behavioural analysis

Video from the OFT was exported and converted from .mp4 to .avi files using Videosolo (https://www.videosolo.com/free-video-converter/), thus enabling viewing in VLC media player (http://www.videolan.org) software. Using videosolo, individual files were trimmed to commence when the bird was placed in camera view, and end after 5 min. Files were uploaded to Ethovision 14.0 (http://www.NOLDUS.com; purchased from and supported by Tracksys: https://www.tracksys.co.uk/) and a protocol was established to measure behaviour. The OFT arena was (virtually) divided into an outer and inner section. The 50 cm^2^ inner section was positioned exactly central to the whole, with a 25 cm border. Latency to move, distance travelled, time spent completely still and time spent in each zone were recorded automatically. Other activity (preening and scratching) was scored manually. Behavioural traits recorded were latencies to move (LtMove, s), visit middle and outer zones (LtVMZ, s; LtVOZ, s), preen (LtPr, s) and scratch (LtScratch, s); distance travelled (cm), velocity (cm/s), number of visits to middle and outer zones (#VtMZ; #VtOZ), time spent in middle and outer zones (TiMZ, s; TiOZ, s), time moving (Tmov, s), and time scratching (TScratch, s).

### Steroid hormone analysis

Steroid hormones were profiled by liquid chromatography mass spectrometry (LC–MS/MS) at the Mass Spectrometry Core, Edinburgh Clinical Research Facility, Centre for Cardiovascular Sciences (QMRI, Little France, Edinburgh), using a low volume adaptation of the method by Denham et al.^62^. Briefly, 100 μL plasma samples collected during the stress trials were aliquoted with 0.005–50 ng calibration standards to a deep 96-well plate enriched with isotopically labelled internal standards (IS) (^13^C_3_-A4, ^13^C_3_-T, d8B; 20µL; 10 ng). They were extracted using an Extrahera liquid handling robot (Biotage, Sweden) transferring to a Supported Liquid Extraction (SLE200) plate, diluting with formic acid (0.1% v/v), and eluting with dichloromethane/isopropanol (98.2 v/v) and reduced to dryness. The extracts were reconstituted in water/methanol (70:30, 100 µL), the plate was sealed and shaken (10 min) before analysis. LC–MS/MS was carried out by injection (20 µL) onto a Kinetex C18 (150 × 3 mm; 2.6 um) column, with a 005 mM ammonium fluoride methanol/water mobile phase system, (0.5 mL/min, 40 °C) on a Shimadzu Nexera uHPLC (Shimadzu, Milton Keynes, UK) interfaced to a QTRAP 6500 + (Sciex, Warrington, UK) mass spectrometer, operated in positive ion electrospray ionisation (ESI) mode at 600 °C, 5.5 kV. Multiple reaction monitoring of steroids and IS were as follows: B (*m/z* 347.1 121.1, 90.9) A (*m/z* 345.1 121.1, 91.2), T (*m/z* 289.1 97.0, 109.2), A4 (*m/z* 287.1 97.0, 78.9), ^13^C_3_T (*m/z* 292.2 100.2), ^13^C_3_A4 (*m/z* 290.2 100.1), d8B (*m/z* 355.3 125.1). Sciex Analyst® 1.6.3 Software was used for instrument control and data acquisition. The peak area ratio of the steroid to internal standard was used to plot a calibration line for each steroid, and least squares regression (1/x weighting) were used to calculate the amounts of steroid.

Baseline and post-stressor, plasma concentrations of corticosterone, 11-dehydrocoricosterone (the inactive form of corticosterone), testosterone, and androsterone (an intermediate in the production of testosterone), were quantified.

### Statistical analysis

Statistical analyses were performed in ASReml^63^ using a simple linear univariate model (y = Xb + ε) for all phenotypic measures except body weight, when a repeated measure mixed linear model (y = Xb + Za + ε) was used, where: y is the vector of observations; b is the vector of fixed effects; a is the vector of permanent environment effects; X and Z are the corresponding incidence matrices; and ε is the vector of residual effects. Fixed effects included parental diet (two-level factor), treatment (two-level factor), age of egg at hatch (a seven-level factor as eggs were collected over a one-week period), sex (male or female), and hatch (a three-level factor used in growth and maturation analyses only), with interactions between fixed effects fitted where appropriate. Egg, hatch, and 12-week weight were fitted as covariates where required, with quail identity fitted as a random effect in the repeated measures model for body weight. Where necessary, the steroid hormone data was natural log (Log_e_) transformed to achieve normal residual distribution, with a constant added where required to transform negative values (this was only applicable to the change in steroid hormone level data). The most parsimonious model for each phenotypic trait was determined by formally testing fixed effects and interactions, and removing those with a significance level above the conditional Wald F-test threshold of 5%. Any observations identified as residual outliers were removed. For the behaviour trial, order of trial was nested within group and day. For the stress response, the pre-stressor hormone plasma concentrations and the change (Δ) between pre- and post-stressor samples (change) was analysed. Male and female hormone plasma concentrations were analysed separately. Multivariate analyses were used to identify between-trait correlation (r) estimates, rescaling (mean-centered/standard deviation) traits to adjust for differences in measurement scale. Significance of r was calculated using the student’s t-test and reported where *p* < 0.05. The data analyst was not blinded to the experimental groups as the complex nature of the statistics involved essentially required exact knowledge of individuals.

### Human and animal rights

Animals were bred and experimental trials were performed at the National Avian Research Facility (NARF) in accordance with the United Kingdom Animal (Scientific procedures) Act 1986, approved by the Roslin Institute ethical review committee under UK Home Office Project Licence number P61FA9171.

## Supplementary Information

Supplementary Information.
