# Supplementary material for: Parental methyl-enhanced diet and in ovo corticosterone affect first generation Japanese quail (Coturnix japonica) development, behaviour and stress response
Source: Sci Rep. 2021 Oct 26;11:21092. doi: 10.1038/s41598-021-99812-w (PMC8548525; doi:10.1038/s41598-021-99812-w)
Supplement: Supplementary file 1 — Supplementary Information. [file 41598_2021_99812_MOESM1_ESM.pdf]

Supplementary table S1. Quail diets.

FORMAT Smutil-4e Ian_Hollows POULTRY GENERAL Page
13:23 17-APR-18 Licence No: 2501

PR TABLE

| Basic Data | | [ 103] | [ 104] |
|---|---|---|---|
| Name : | | QUAIL BREEDER/LAYER | QUAIL STARTER |
| Raw Material | | % | % |
| 2 MAIZE | * | 25.0000 | 25.0000 |
| 10 WHEAT | * | 25.0000 | 25.0000 |
| 210 PRAIRIE | * | 10.0000 | 6.0000 |
| 424 SOYA EXT HIPRO FINE | * | 20.0000 | 20.0000 |
| 425 FULL FAT SOYA MASHAM | * | 7.5000 | 10.0000 |
| 459 PROVIMI 66 FISH | * | 2.5000 | 10.0000 |
| 701 MONOCALCIUM PHOSPHATE | * | 0.5000 | 0.0000 |
| 703 LIMESTONE GRANULES | * | 5.0000 | 0.0000 |
| 713 L LYSINE HCL | * | 0.1000 | 0.0000 |
| 802 SOYA BEAN OIL | * | 1.8000 | 1.5000 |
| 904 SALT | * | 0.1000 | 0.0000 |
| 3489 QUAIL STARTER BREEDER | * | 2.5000 | 2.5000 |
| Total: | | 100.0000 | 100.0000 |

| Nutrient | Analysis | Analysis |
|---|---|---|
| OIL EE : | 5.0195 | 5.5300 |
| PROTEIN : | 24.4948 | 27.7750 |
| FIBRE : | 2.5925 | 2.7030 |
| TLYSINE : | 1.4157 | 1.7090 |
| AVLYSINE : | 1.3395 | 1.6044 |
| METH : | 0.5258 | 0.5910 |
| M+C : | 0.9198 | 1.0010 |
| THREO : | 0.9068 | 1.0645 |
| TRYPT : | 0.2367 | 0.2824 |
| CALCIUM : | 2.6187 | 0.9892 |
| PHOS : | 0.7448 | 0.8512 |
| AVPHOS : | 0.5178 | 0.6258 |
| SALT : | 0.3840 | 0.4335 |
| SODIUM : | 0.1844 | 0.1991 |
| ASH : | 10.4300 | 6.2730 |
| ME-P : | 12.4385 | 12.9595 |
| VIT A : | 13.5000 | 13.5000 |
| VIT D3 : | 5.0000 | 5.0000 |
| VIT E : | 100.0000 | 100.0000 |

TARGET FEEDS LIMITED
Feed formulations from Ian Hollows...
Wood Farm, Coppice Lane, Coton, Whitchurch, Shropshire. SY13 3LT
Tel: 01948 880 598 Fax: 01948 880730 Email: ian@targetfeeds.com Website: www.targetfeeeds.com
UFAS Universal Feed Assurance Scheme
Registered in England
Company No. 2194571

PR TABLE

****************

| Basic Data | | [3489] |
|---|---|---|
| Name | : | QUAIL STARTER BREEDER |

| Raw Material | | % |
|---|---|---|
| 218 SIEVED SOYA HULL MEAL | * | 3.5000 |
| 712 LYSINE BATCH: | * | 2.0000 |
| 713 METHIONINE BATCH: | * | 0.5000 |
| 900 LIMESTONE TRUCAL 52 GRAN | * | 4.5812 |
| 902 MCP GRANULES | * | 8.0000 |
| 904 SALT | * | 2.0000 |
| 906 SODIUM BICARBONATE | * | 0.7500 |
| 9021 VITAMIN D3 500 BATCH: | * | 0.0040 |
| 9022 VITAMIN E 50% BATCH: | * | 0.1500 |
| 9028 CHOLINE CHL.50% BATCH: | * | 1.0000 |
| 9030 BIOTIN 2% BATCH: | * | 0.0038 |
| 9036 FOLIC ACID BATCH: | * | 0.0010 |
| 9044 SELENIUM PREMIX 1.0% TFL | * | 0.0100 |
| 9142 POULTRY 1 TE BATCH: | * | 2.5000 |
| Total: | | 25.0000 |

| Nutrient | | Analysis |
|---|---|---|
| USAGE | : | 25.0000 |
| VIT A | : | 10.0000 |
| VIT D3 | : | 5.0000 |
| VIT E | : | 100.0000 |
| VIT B1 | : | 3.0000 |
| VIT B2 | : | 10.0000 |
| VIT B6 | : | 3.0000 |
| VIT B12 | : | 30.0000 |
| HETRA | : | 5.0000 |
| NICO | : | 60.0000 |
| PANTO | : | 15.0000 |
| FOLIC | : | 2.5000 |
| BIOTIN | : | 201.0000 |
| CHOLCHL | : | 500.0000 |
| FE | : | 20.0000 |
| MN | : | 100.0000 |
| CU | : | 10.0000 |
| ZN | : | 80.0000 |
| I | : | 1.0000 |
| SE | : | 0.3000 |
| LYSHCL | : | 2000.0000 |
| METH | : | 500.6650 |
| *CA/USA | : | 15.8292 |
| *PHO/USA | : | 7.2892 |
| *SAL/USA | : | 8.0063 |
| *SOD/USA | : | 4.0275 |

TARGET FEEDS LIMITED
Feed formulations from Ian Hollows...
Wood Farm, Coppice Lane, Coton, Whitchurch, Shropshire. SY13 3LT
Tel: 01948 880 598 Fax: 01948 880730 Email: ian@targetfeeds.com Website: www.targetfeeeds.com
UFAS Universal Feed Assurance Scheme
Registered in England
Company No. 2194571

Supplementary Table S2. Quail rearing facility pen and environmental details for all quail generations.

| *AGE (DAYS)* | *PEN SIZE ($M^2$)* | *DAYLIGHT (HRS)* | *TEMPERATURE ($^OC$)* |
|---|---|---|---|
| ***1-3*** | 1 | 18 | 27 |
| ***4-14*** | 2 | 14 | 27 |
| ***15-21*** | 4 | 14 | 27 |
| ***21+*** | 8 | 10 | 25 |
| ***42*** | 8 | 14 | 22 |

Supplementary Table S3. Predicted mean values of diet, diet by treatment interactions (diet*treatment), and sex, (± s.e.m. and significance (*p*) values) for parental ($G_0$) and first generation ($G_1$) quail: wt = weight; Control = control diet; HiBET = betaine enhanced diet; vehicle = control *in ovo* treatment. *Figures in italics fell just short of significance.*

| | **DIET** | | | **DIET*IN OVO TREATMENT** | | | | | **SEX** | | |
|---|---|---|---|---|---|---|---|---|---|---|---|
| **CO-VARIATE/FIXED EFFECT** | **Control** | **HiBET** | ***P*** | **Control* Vehicle** | **Control* Corticosterone** | **HiBET* Vehicle** | **HiBET* Corticosterone** | ***P*** | **Male** | **Female** | ***P*** |
| ***$G_0$ BODY WT (G)*** | | | | | | | | | 225.8 (9.48) | 290.1 (5.53) | <0.001 |
| ***$G_0$ LIVER WT (G)*** | | | | | | | | | 4.11 (0.29) | 6.05 (0.15) | <0.001 |
| ***EGG WT (G)*** | 13.41 (0.09) | *13.76 (0.10)* | 0.009 | | | | | | | | |
| ***$G_1$ HATCH WT (G)*** | 9.41 (0.08) | 9.13 (0.08) | <0.001 | | | | | | 9.19 (0.07) | 9.35 (0.8) | 0.015 |
| ***$G_1$ 12WK WT (G)*** | | | | | | | | | 167.3 (1.66) | 178.7 (1.70) | <0.001 |
| ***$G_1$ OVIDUCT WT (G)*** | 5.43 (0.52) | 7.93 (0.55) | 0.001 | | | | | | | | |
| ***YYF (#)*** | 3.00 (0.30) | 4.23 (0.33) | 0.007 | | | | | | | | |
| ***$G_1$ OVARY WT (G)*** | *0.68 (0.04)* | *0.81 (0.04)* | *0.041* | | | | | | | | |
| ***$G_1$ SPLEEN WT (G)*** | | | | | | | | | 0.14 (0.01) | 0.16 (0.01) | 0.048 |
| ***$G_1$ LIVER WT (G)*** | *4.03 (0.09)* | *4.27 (0.99)* | *0.065* | 4.15 (0.12) | 3.91 (0.13) | 4.13 (0.15) | 4.42 (0.14) | 0.048 | 3.49 (0.10) | 4.63 (0.09) | <0.001 |

Supplementary Table S4. Estimated between-trait correlation matrix (± s.e.m.). All traits refer to weights (wt) except for number of yellow yolk follicles (YYF #). Significant values (*p*<0.05) are presented in bold text.

| | EGG WEIGHT | $G_1$ HATCH WT | $G_1$ 12 WEEK WT | $G_1$ TESTES WT | $G_1$ OVIDUCT WT | YYF # | $G_1$ OVARY WT | $G_1$ SPLEEN WT |
|---|---|---|---|---|---|---|---|---|
| **$G_1$ HATCH WT** | **0.75 (0.03)** | | | | | | | |
| **$G_1$ 12 WEEK WT** | **0.22 (0.07)** | **0.24 (0.07)** | | | | | | |
| **$G_1$ TESTES WT** | 0.11 (0.10) | 0.08 (0.10) | 0.03 (0.10) | | | | | |
| **$G_1$ OVIDUCT WT** | 0.00 (0.10) | 0.12 (0.10) | **0.27 (0.10)** | | | | | |
| **YYF #** | 0.02 (0.10) | 0.11 (0.10) | 0.16 (0.10) | | **0.83 (0.03)** | | | |
| **$G_1$ OVARY WT** | 0.09 (0.10) | **0.19 (0.10)** | **0.31 (0.09)** | | **0.66 (0.06)** | **0.65 (0.06)** | | |
| **$G_1$ SPLEEN WT** | **0.23 (0.07)** | **0.20 (0.07)** | **0.27 (0.07)** | **0.27 (0.10)** | **0.23 (0.09)** | -0.05 (0.11) | **0.25 (0.09)** | |
| **$G_1$ LIVER WT** | **0.15 (0.07)** | **0.13 (0.07)** | **0.63 (0.04)** | -0.11 (0.10) | 0.00 (0.11) | 0.01 (0.11) | 0.13 (0.11) | **0.22 (0.07)** |

Supplementary Table S5. Productivity by $G_1$ group, indicated by mean numbers of yellow yolk follicles (YYF) present in oviduct, and uterine egg presence at 12 weeks of age. Diet/treatment key: - = no diet or *in ovo* treatment applied, + = diet or *in ovo* treatment applied; # = number; Out of lay = no YYF or egg present; % = percentage.

| GROUP | -/- | -/+ | +/- | +/+ |
|---|---|---|---|---|
| **FEMALES (#)** | 28 | 23 | 16 | 28 |
| **OUT OF LAY (#)** | 9 | 9 | 1 | 2 |
| **OUT OF LAY (%)** | 32.1 | 39.0 | 6.3 | 7.1 |
| **YYF (#)** | 3.2 | 2.8 | 4.5 | 4.4 |
| **EGG PRESENCE (#)** | 0.5 | 0.6 | 0.6 | 0.8 |
| **MEAN YIELD/BIRD** | 3.68 | 3.35 | 5.13 | 5.11 |

Supplementary Table S6. Predicted mean values of diet, treatment, and sex (± s.e.m. and significance (*p*) values) for $Hatch_1$ quail. Logged values are indicated. (Control = control diet / *in ovo* treatment; HiBET = betaine enhanced diet; s = seconds, cm = centimetre; # = number).

| | **DIET** | | | ***IN OVO* TREATMENT** | | | **SEX** | | |
|---|---|---|---|---|---|---|---|---|---|
| **TRAIT** | **Control** | **HiBET** | ***P*** | **Control** | **Corticosterone** | ***P*** | ***Male*** | **Female** | ***P*** |
| **LATENCY TO MOVE (*Log*$_e$, S)** | -2.07 (0.42) | -2.77 (0.37) | 0.019 | | | | | | |
| **LATENCY TO VISIT MIDDLE (*Log*$_e$, S)** | | | | -0.28 (0.75) | -0.1.53 (0.73) | 0.038 | | | |
| **VISITS TO MIDDLE (#)** | | | | | | | 7.61 (1.49) | 4.20 (1.79) | 0.015 |
| **LATENCY TO VISIT OUTER ZONE (*Log*$_e$, S)** | | | | | | | -2.58 (0.74) | -0.86 (0.88) | 0.013 |
| **VISITS TO OUTER ZONE (#)** | | | | | | | 8.01 (1.68) | 4.86 (2.01) | 0.043 |
| **TIME IN OUTER ZONE (*Log*$_e$, S)** | | | | | | | 265.7 (22.9) | 221.5 (26.2) | 0.029 |
| **DISTANCE TRAVELLED (CM)** | | | | | | | 6.66 (0.23) | 6.16 (0.27) | 0.019 |
| **VELOCITY (*Log*$_e$, CM/S)** | | | | | | | 0.94 (0.23) | 0.52 (0.27) | 0.043 |
| **TIME MOVING (S)** | | | | | | | 93.08 (15.9) | 62.0 (19.0) | 0.031 |
| **LATENCY TO SCRATCH (*Log*$_e$, S)** | | | | | | | 4.12 (0.23) | 4.61 (0.27) | 0.034 |
| **TIME SCRATCHING (S)** | | | | | | | 114.1 (19.0) | 73.8 (22.4) | 0.033 |

Supplementary Table S7. Estimated correlations (± s.e.m.) between behaviour and stress traits. All significant values (*p*<0.05) are presented in bold text. (# = number; Δ = change in levels).

| | **# VISITS TO MIDDLE ZONE** | **DISTANCE TRAVELLED** | **VELOCITY** | **TIME MOVING** | **BASELINE CORTICOSTERONE** | **BASELINE TESTOSTERONE** | **BASELINE ANDROSTENEDIONE** |
|---|---|---|---|---|---|---|---|
| **DISTANCE TRAVELLED** | **0.82 (0.04)** | | | | | | |
| **VELOCITY** | **0.80 (0.04)** | **0.98 (0).00** | | | | | |
| **TIME MOVING** | **0.59 (0.08)** | **0.79 (0.04)** | **0.79 (0.05)** | | | | |
| **BASELINE CORTICOSTERONE** | -0.09 (0.12) | -0.11 (0.12) | -0.10 (0.12) | -0.04 (0.12) | | | |
| **BASELINE TESTOSTERONE** | -0.02 (0.12) | **-0.24 (0.12)** | **-0.24 (0.12)** | -0.09 (0.12) | **0.25 (0.12)** | | |
| **BASELINE ANDROSTENEDIONE** | -0.08 (0.12) | -0.16 (0.12) | -0.15 (0.12) | -0.20 (0.12) | 0.15 (0.12) | **0.32 (0.11)** | |
| **Δ_ANDROSTENEDIONE** | 0.03 (0.12) | 0.12 (0.12) | 0.10 (0.12) | 0.15 (0.12) | -0.12 (0.12) | **-0.25 (0.12)** | **-0.95 (0.01)** |

Supplementary Table S8. Estimated effect sizes of diet, treatment, and diet by treatment interactions, (diet*treatment) ± s.e.m. and significance (*p*) values of baseline and stress induced changes (Δ) in steroid plasma concentrations of $G_1$ $Hatch_1$ quail. All effect sizes are the difference from control fed and control *in ovo* treatments. HiBET = betaine enhanced diet.

| | | **DIET** | | | ***IN OVO* TREATMENT** | | | **DIET*TREATMENT** | |
|---|---|---|---|---|---|---|---|---|---|
| **TRAIT** | **Sex** | **HiBET** | ***P*** | **Sex** | **Corticosterone** | ***P*** | **Sex** | **HiBET* Corticosterone** | ***P*** |
| **BASELINE 11-DEHYDROCORTICOSTERONE (*$Log_e$*, ng/ml)** | Male | -0.47 (0.12) | <0.001 | | | | | | |
| **BASELINE CORTICOSTERONE (*$Log_e$*, ng/ml)** | Male | | | | | | Male | -0.89 (0.23) | 0.002 |
| **BASELINE ANDROSTENEDIONE** | Female | 0.13 (0.04) | 0.002 | Male | 0.51 (0.14) | <0.001 | | | |
| **Δ_ANDROSTENEDIONE (*$Log_e$*, ng/ml)** | Male | | | | 1.14 (0.05) | 0.024 | | | |
| **BASELINE TESTOSTERONE (*$Log_e$*, ng/ml)** | Male | | | | 0.79 (0.32) | 0.021 | | | |
